# Two new Brazilian species of *Loxosceles* Heinecken & Lowe, 1832 with remarks on *amazonica* and *rufescens* groups (Araneae, Sicariidae)

**DOI:** 10.3897/zookeys.667.11369

**Published:** 2017-04-10

**Authors:** Caroline Sayuri Fukushima, Rute Maria Gonçalves de Andrade, Rogério Bertani

**Affiliations:** 1 Laboratório Especial de Ecologia e Evolução, Instituto Butantan, Av. Vital Brazil, 1500 CEP 05503-900, São Paulo, São Paulo, Brazil; 2 Fundação Museu do Homem Americano, Laboratório de Vestígios Orgânicos, Centro Cultural Sérgio Motta s/n, CEP 64770-000, Caixa Postal 2, São Raimundo Nonato, Piauí, Brazil

**Keywords:** Alagoas, Brown recluse spider, Caatinga, Cave, Rio Grande do Norte

## Abstract

The genus *Loxosceles* Heinecken & Lowe, 1832 has 91 representatives in the New World. Despite medical relevancy, the taxonomy of the genus is poorly understood. South American *Loxosceles* were divided into four groups of species: *laeta*, *spadicea*, *gaucho* and *amazonica*; this last one has a single species, *Loxosceles amazonica* Gertsch, 1967. More recently, the natural occurrence of *L. amazonica* in the New World has been questioned, due to the strong morphological resemblance and close phylogenetic relationship with Old World species, mainly with *Loxosceles rufescens* (Dufour, 1820). Herein, *L. amazonica* is rediagnosed and its morphological variation and natural distribution discussed. Two new species closely related to it from northeastern Brazil are also described, *Loxosceles willianilsoni* **sp. n.**, from the state of Rio Grande do Norte, and *Loxosceles muriciensis* **sp. n.**, from the state of Alagoas. The relationships of these new species with *L. amazonica* and *L. rufescens* are discussed.

## Introduction

*Loxosceles* Heinecken & Lowe, 1832 is a speciose spider genus with a core distribution in the New World (World Spider Catalog 2016). Several species are known also from Africa, Middle East, Mediterranean Europe and two species from China were recently described (World Spider Catalog 2016). Many species were reported as causing bites of importance to human health and several studies on their venom have been published (Gertsch 1967, Tambourgi et al. 2000, Isbister and Fan 2011). Despite this, the taxonomy of the genus is poorly understood. The most comprehensive works were done by Gertsch (1958, 1967) and Gertsch and Ennik (1983) who revised New World species. After these revisions, other species were sporadically described and more recently the African, Middle East and Asian species received more attention (Binford et al. 2008, Duncan et al. 2010, Lotz 2012, Planas and Ribera 2015, Wang 1994).

The South American *Loxosceles* were revised by Gertsch (1967), who created four groups of species: *laeta* with 26 species, *spadicea* with three species, *gaucho* with six species and *amazonica* with a single species. *Loxosceles amazonica* Gertsch, 1967 has been recorded from localities in the Amazon in Brazil, and Peru to northeastern Brazil. More recently, the natural distribution in the New World has been questioned, due to the strong morphological resemblance to the Old World species, mainly with *Loxosceles rufescens* (Dufour, 1820) (Binford et al. 2008; Duncan et al. 2010). Molecular analyses has also retrieved *L. amazonica* to be closely related to the Old World species (Binford et al. 2008; Duncan et al. 2010), therefore, *L. amazonica* origin and its relationship is still up for debate.

Herein, we describe two new species closely related to *L. amazonica* from northeastern Brazil. The relationship of these new species with *L. amazonica* and *L. rufescens* is discussed.

## Materials and methods

The general format of the description follows Gertsch (1967). All measurements are in millimeters. Measurements of the legs and palp were taken from the dorsal aspect of the left side (unless appendages were lost or obviously regenerated) with a Mitutoyo^®^ digital caliper, which had an error of 0.005 mm, rounded up to two significant decimals. Structures from the left side of the specimens were chosen for descriptions. When using structures from the right side, the figures were mirrored to show them as coming from the left side and allowing easy comparison. The copulatory organs of females were dissected and submitted to digestion by a commercial protein remover for contact lenses (with pancreatin) during some minutes in order to observe the internal structure; when necessary, they were also cleared with clove oil. A Leica LAS Montage and LAS 3D module mounted on a Leica M205C dissecting microscope were used for image capture and measurements of other spider structures.

### Abbreviations

**ALE** anterior lateral eye,

**ESEC** Ecological Station,

**FLONA** National Forest,

**PARNA** National Park,

**PLE** posterior lateral eye,

**PME** posterior median eye.

The examined specimens are deposited at **MNRJ**, Museu Nacional, Rio de Janeiro, and **AMNH**, American Museum of Natural History, New York. Geographical coordinates are denoted as primary sources between round brackets, secondary sources (Google Earth) between square brackets. The coordinates from the secondary source were obtained from the center of the municipality cited in the specimen label and are in DMS (Degrees, Minutes and Seconds) format rounded off to minutes. Maps were made with SimpleMappr, an online tool used to produce maps (Shorthouse 2010).

## Taxonomy

### Loxosceles amazonica

Taxon classificationAnimaliaAraneaeSicariidae

Gertsch, 1967

Figs 1–6 , 7–13 , 14–17 , 18–21 , 22–25 , 26–31 , 32–35 , 36–39 , 40–47 , 48–51 , 78–79

Loxosceles amazonica Gertsch, 1967: 143, pl. 4, figs 7−10, pl. 5, figs 6−7 (female holotype examined (AMNH), Brazil, state of Mato Grosso, Santa Isabel, Araguaia river, Mato Grosso side, 15–25 July 1957, B. Malkin col., receptacles not in the vial); Lucas, Cardoso and Moraes 1986: 130, figs 3−4; Duncan et al. 2010: 241, fig. 3; World Spider Catalog 2016.

#### Material examined

**(Table 3).** BRAZIL: *Piauí*, Serra Branca, Parque Nacional Serra da Capivara, São Raimundo Nonato [9°00'S, 42°41'W], 1 male, 1 female and 11 immatures, R. M. Gonçalves Andrade col. (MNRJ 6927); *Rio Grande do Norte*: Serra Negra do Norte, ESEC Seridó (6°34'S, 37°15'W), 2 females and 5 males, C. S. Fukushima, K. C. T. Riciluca and N. M. Gonçalves col., 14 March 2014, ref. Ser 8, 12, 2, 7, 9, 10, 33, respectively (MNRJ 6928); 1 female, under tree bark, during the night, C. S. Fukushima col., 14 March 2014, ref. C28 (MNRJ 6929); 1 female, C. S. Fukushima col., 14 March 2014, inside tree trunk, during the day, ref. C44 (MNRJ 6930); 1 male, C. S. Fukushima col., 14 March 2014, ref. C41 (MNRJ 7303); Açu, FLONA de Açu (5°34'S, 36°56'W), 1 female, under old house debris, during the night, L. Monteiro col., 30 October 2014, ref. L72 (MNRJ 6931); 1 female, under tree bark, during the day, C. S. Fukushima col., 30 October 2014, ref. C599 (MNRJ 6932); 1 female, near Carnaúba trees, during the day, K. C. T. Riciluca col., 26 March 2014, ref. K137 (MNRJ 6933); 1 female, in a vacated old house during the night, C. S. Fukushima col., 23 March 2014, ref. C163 (MNRJ 6934); 1 male, under roof tiles, C. S. Fukushima col., 23 March 2014, ref. C167g (MNRJ 6935); 1 male, under roof tiles, C. S. Fukushima col., 23 March 2014, ref. C167o (MNRJ 6936); 1 male, under roof tiles, C. S. Fukushima col., 30 October 2014, ref. C631 (MNRJ 6937); 1 male, in fallen Carnaúba tree, during the night, N. M. Gonçalves col., 25 March 2014, ref. N186 (MNRJ 6938); 1 male, under roof tiles, during the night, C. S. Fukushima col., 23 March 2014, ref. XXXI (MNRJ 6939); 1 female, K. C. T. Riciluca col., March 2014, ref. K133 (MNRJ 7305); Martins (6°04'S, 37°54'W), 1 female, Mirante-Casa de Pedra cave track, during the night, C. S. Fukushima col., 20 March 2014, ref. C144 (MNRJ 6940); 1 female, near Casa de Pedra cave, during the day, N. M. Gonçalves col., 19 March 2014, ref. N81 (MNRJ 6941); 1 female, Mirante-Casa de Pedra cave track, during the day, N. M. Gonçalves col., 20 March 2014, ref. N91 (MNRJ 6942); 1 female, under fallen tree, near grange of Sr. Clesinho, during the day, A. P. L. Giupponi col., 23 October 2014, ref. A132 (MNRJ 6943), 1 female, near Casa de Pedra cave, under rock, during the night, C. S. Fukushima col., 23 October 2014, ref. C495 (MNRJ 6944); 1 male, in a ravine near Casa de Pedra cave, during the night, K. C. T. Riciluca col., 19 March 2014, ref. K59 (MNRJ 6945); 1 male, near Casa de Pedra cave, during the day, C. S. Fukushima col., 19 March 2014, ref. C103 (MNRJ 6946); 1 male, in a ravine, C. S. Fukushima col., 19 March 2014, ref. C116 (MNRJ 6947); 1 female, near Casa de Pedra cave, C. S. Fukushima col., 23 October 2014, ref. C497; 1 male, Mirante-Casa de Pedra cave track, C. S. Fukushima col., 20 March 2014, ref. C148 (MNRJ 7306); Macaíba, Escola Agrícola de Jundiaí (5°53'S, 35°21'W), 1 male (MNRJ 6948) and 1 female (MNRJ 6949), in a tree trunk during the night, C. S. Fukushima and W. Pessoa col., 13 September 2013 (ref. AV046, AV047, respectively); *Ceará*, Santa Quitéria (4°19'S, 40°09'W), 1 male and 1 immature male, D. R. Pedroso col., 3–12 February 2014 (MNRJ 6950); 1 male, 1 female and 9 immatures, Gruta W13, SAD’69, Camp 1, F. Pellegatti & D. R. Pedroso col., 3–13 February 2014 (MNRJ 6952).

#### Diagnosis.

**Figures 1–6.**
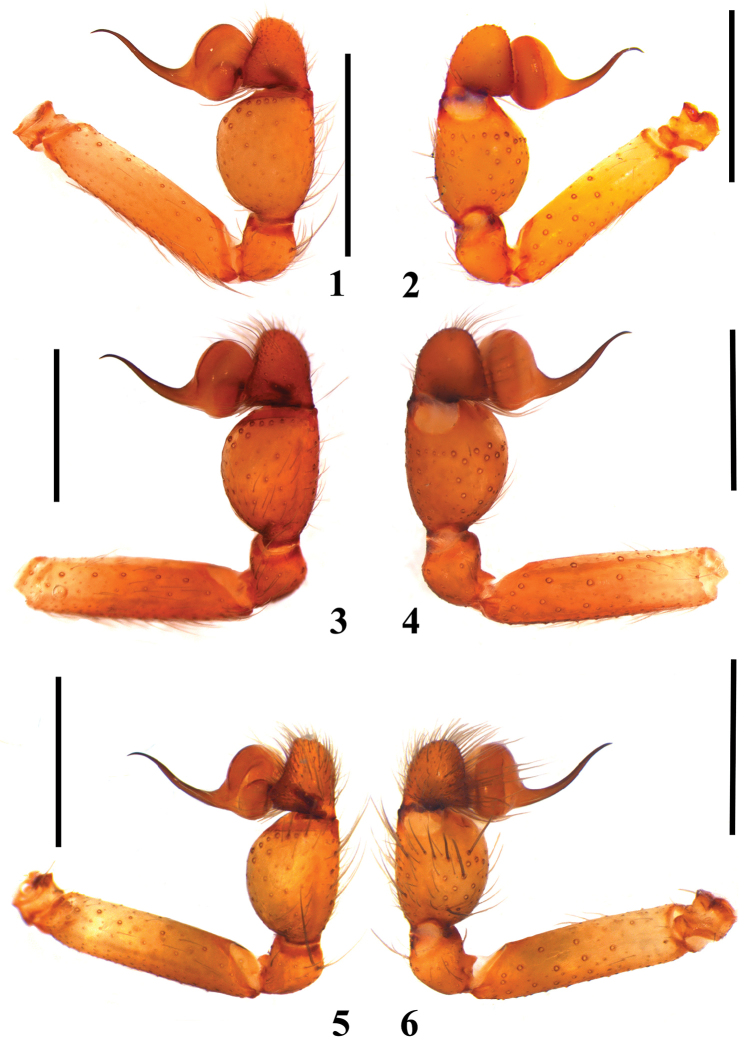
*Loxosceles amazonica*, male palpal bulbs. **1–2** Serra Negra do Norte, ESEC Seridó, state of Rio Grande do Norte, Brazil (MNRJ 6928, ref. Ser 7), left palp. **1** retrolateral **2** prolateral **3–4** Açu, FLONA de Açu, state of Rio Grande do Norte, Brazil (MNRJ 6939), left palp **3** retrolateral **4** prolateral **5–6** Martins, state of Rio Grande do Norte, Brazil (MNRJ 7306), right palp (mirrored) **5** retrolateral **6** prolateral. Scale bars: 1mm.

Males of *L. amazonica* resemble those of *Loxosceles rufescens*, *Loxosceles bentejui* Planas & Ribera, 2015, *Loxosceles foutadjalloni* Millot, 1941, *Loxosceles guayota* Planas & Ribera, 2015, *Loxosceles hupalupa* Planas & Ribera, 2015, *Loxosceles lacta* Wang, 1994, *Loxosceles mahan* Planas & Ribera, 2015, *Loxosceles tazarte* Planas & Ribera, 2015, *Loxosceles tibicena* Planas & Ribera, 2015, *Loxosceles willianilsoni* sp. n., and *Loxosceles muriciensis* sp. n. by incrassated palpal tibia, longer than cymbium (Figs 1–2). They differ from those of *L. hupalupa*, *L. mahan* and *L. tazarte* by having shorter embolus (Figs 1–2), and entire pars cephalica as well as carapace border dark brown (Fig. 14), best seen in live specimens. From those of *L. rufescens*, *L. bentejui*, *L. foutadjalloni*, *L. guayota*, *L. lacta*, *L. tibicena*, *L. willianilsoni* sp. n. and *L. muriciensis* sp. n., they can be distinguished by having embolus with a mild retrolateral curvature along its length (Fig. 11). Females of *L. amazonica* resemble those of *L. rufescens*, *L. bentejui*, *L. foutadjalloni*, *L. hupalupa*, *L. lacta*, *L. mahan*, *L. tazarte*, *L. tibicena*, *L. willianilsoni* sp. n. and *L. muriciensis* sp. n. by having spermathecae with large seminal receptacles and dark sclerotized lateral bands (Fig. 26). Females of *L. amazonica* can be distinguished from all these species by a cluster of globular lobes on apex of seminal receptacles (Figs 26–31). Additionally, *L. amazonica* males and females can be distinguished from *L. mahan*, *L. tazarte*, *L. bentejui*, *L. guayota*, *L. tibicena* and *L. hupalupa* by lacking a conspicuous dark V-mark posteriorly on pars cephalica.

#### Natural history.

Despite its specific epithet, *L. amazonica* specimens were found in areas covered by caatinga (Figs 36–47), a semi-arid vegetation found in northeastern Brazil (Fig. 78). At FLONA de Açu, specimens were found under rocks and tree bark, and also under or inside fallen trees, especially carnaúbas (*Copernicia prunifera* Miller) (Figs 36–39). They were also found at vacant old houses inside an area of conservation unit, and under house debris near the FLONA’s base.

The ESEC Seridó is located on a *sui generis* region of the state of Rio Grande do Norte characterized by a hyper-xerophilous, arboreal-shrubby caatinga, with irregular precipitation of 500 to 800 mm/year (Varella-Freire 2002). Specimens of *L. amazonica* were found throughout different landscapes of the ESEC (Figs 40–43). They were found under rocks and tree bark in shaded areas (Fig. 44), inside termite nests (Fig. 47) or cracks of rocky outcrops (Fig. 45), under fallen trees (Fig. 46) or under house debris near ESEC’s base.

Specimens of *L. amazonica* were also found in Martins, state of Rio Grande do Norte, “a brejo de altitude” region, i.e. an area covered by humid forest surrounded by arid caatinga (Pereira Filho and Montingelli 2011), usually over mountains and hillsides with an elevation of more than 500 m (Ruiz-Esparza 2009) and that receives more than 1,200 mm of orographic rains (Prado 2003, in Ruiz-Esparza 2009). We found specimens of *L. amazonica* in ravines near the town (Fig. 48), in a trail on the top on the hill (Fig. 49) and under old house debris close to more humid and higher areas (about 700 m a.s.l.) (Fig. 50), as well as under rocks and tree bark near Casa de Pedra cave, in a lower region with caatinga vegetation (about 300 m a.s.l.) (Fig. 51). No specimens were found inside Casa de Pedra cave.

#### Spermatheca variation

**(see Fig. 79).** Specimens vary in number and size of globular lobes on spermatheca apex and seminal receptacles proportions. Specimens from Martins and Macaíba in the State of Rio Grande do Norte (Figs 26 and 31, respectively), São Raimundo Nonato, state of Piauí (Fig. 27) and Santa Quitéria, state of Ceará (Fig. 30) have three to six lobes in each spermatheca, more or less similar in size. The seminal receptacles of specimens of these areas are slightly short and trapezoid. On the other hand, specimens of ESEC Seridó and FLONA de Açu, both in the state of Rio Grande do Norte (Figs 28 and 29, respectively) have four to five lobes, usually one of them larger than the others. The seminal receptacles are slightly longer, with a triangular shape.

It is not clear how these genitalic traits vary along the distribution of *L. amazonica* or if these variations reflect a higher diversity in *amazonica* lineage. Variation in the morphology of palps and spermatheca of other *Loxosceles* species has already been noted, such as in *L. rufescens* (Brignoli 1969). However, Duncan et al. (2010) recovered a monophyletic group of specimens that morphologically resemble *L. rufescens*, within which there are divergent clusters of specimens and populations, but with genetic distances high enough to be considered as cryptic species. In the same way, the slight morphological variations in *L. amazonica* could correspond to separated species, only detectable through a molecular approach, which was beyond the scope of this study.

**Figures 7–13.**
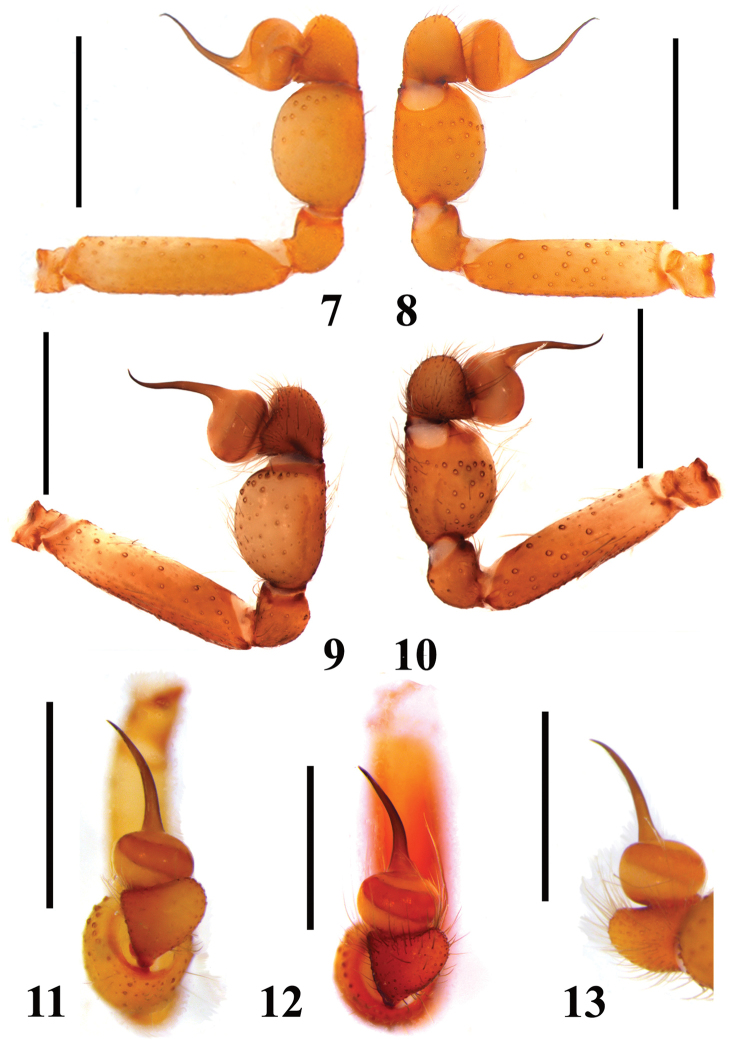
*Loxosceles amazonica*, male palpal bulbs, left palp. **7–8** Santa Quitéria, state of Ceará, Brazil (MNRJ 6950) **7** retrolateral **8** prolateral **9–10** São Raimundo Nonato, state of Piauí, Brazil (MNRJ 6927, ref. GSB11A-17) **9** retrolateral **10** prolateral. **11–13** dorsal **11** Açu, FLONA de Açu, state of Rio Grande do Norte, Brazil (MNRJ 6936) **12** Serra Negra do Norte, ESEC Seridó, state of Rio Grande do Norte, Brazil (MNRJ 6928, ref. Ser 7) **13** Santa Quitéria, state of Ceará, Brazil (MNRJ 6950). Scale bars: 1mm.

**Figures 14–17.**
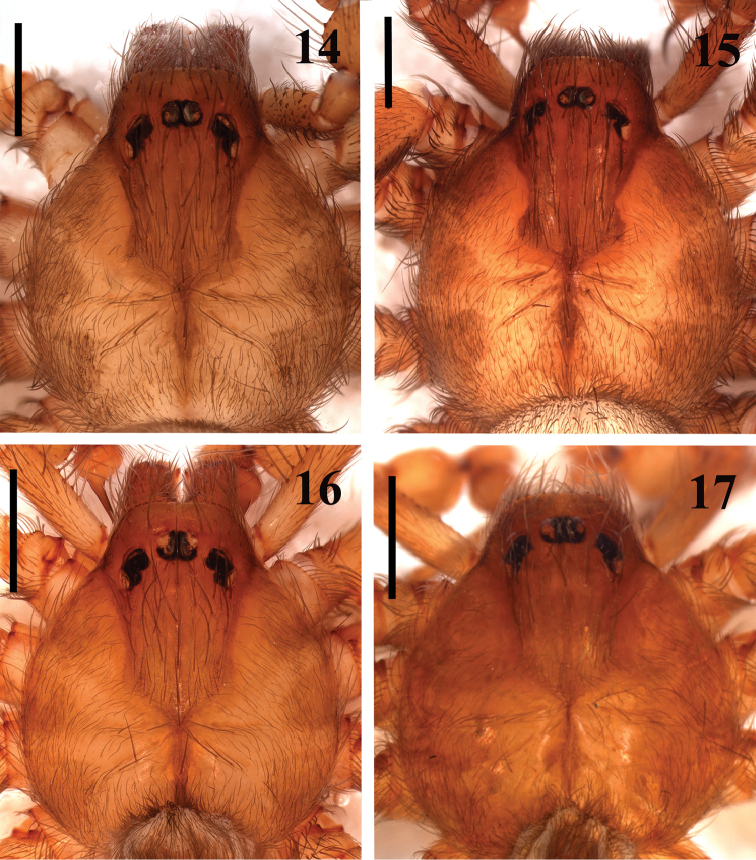
*Loxosceles amazonica*, male carapace. **14–15** Açu, FLONA de Açu, state of Rio Grande do Norte, Brazil **14** MNRJ 6935 **15** MNRJ 6936 **16** Serra Negra do Norte, ESEC Seridó, state of Rio Grande do Norte, Brazil (MNRJ 6928, ref. Ser 7) **17** Martins, state of Rio Grande do Norte, Brazil (MNRJ 6947). Scale bars: 1mm.

**Figures 18–21.**
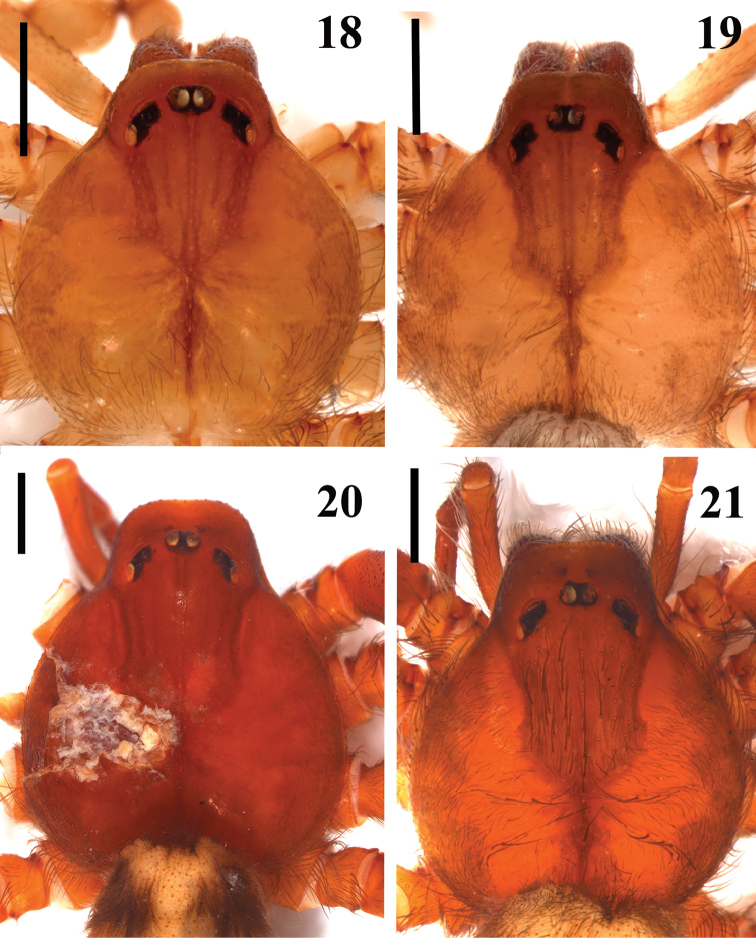
*Loxosceles amazonica*, carapace. **18–19** Male **18** São Raimundo Nonato, state of Piauí, Brazil (MNRJ 6927, ref. GSB11A-17) **19** Santa Quitéria, state of Ceará, Brazil (MNRJ 6950) **20–21** Female **20** holotype, Santa Isabel, state of Mato Grosso, Brazil (AMNH) **21** Açu, FLONA de Açu, state of Rio Grande do Norte, Brazil (MNRJ 7305). Scale bars: 1mm.

**Figures 22–25.**
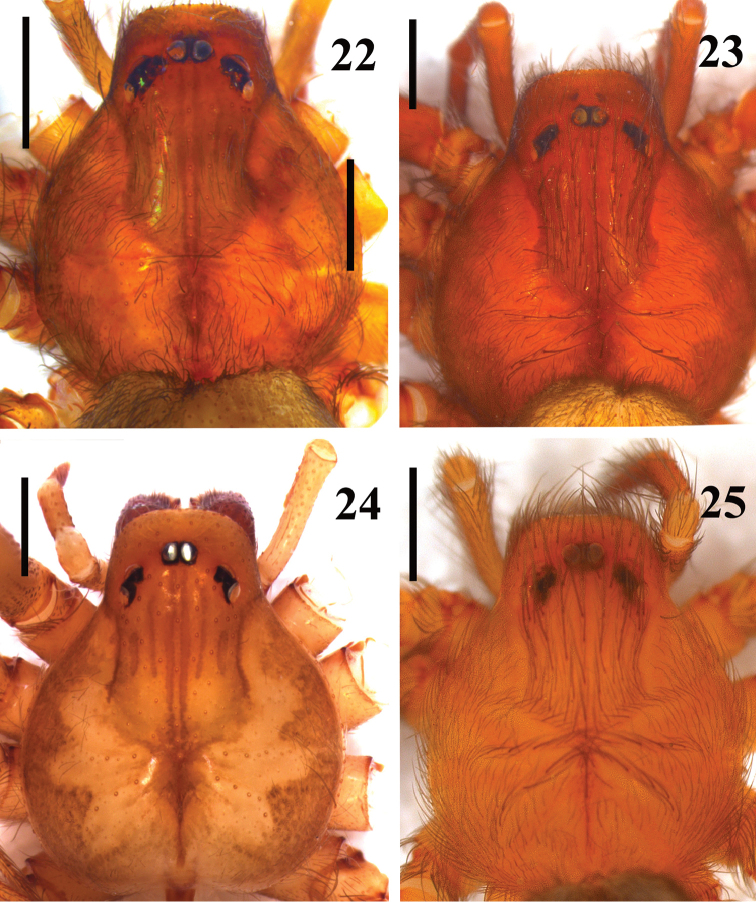
*Loxosceles amazonica*, carapace, female. **22** Martins, state of Rio Grande do Norte, Brazil (MNRJ 7304) **23** Serra Negra do Norte, ESEC Seridó, state of Rio Grande do Norte, Brazil (MNRJ 6928, ref. Ser 8) **24** Santa Quitéria, state of Ceará, Brazil (MNRJ 6952) **25** São Raimundo Nonato, state of Piauí, Brazil (MNRJ 6927, ref. GSB11A-17). Scale bars: 1mm.

**Figures 26–31.**
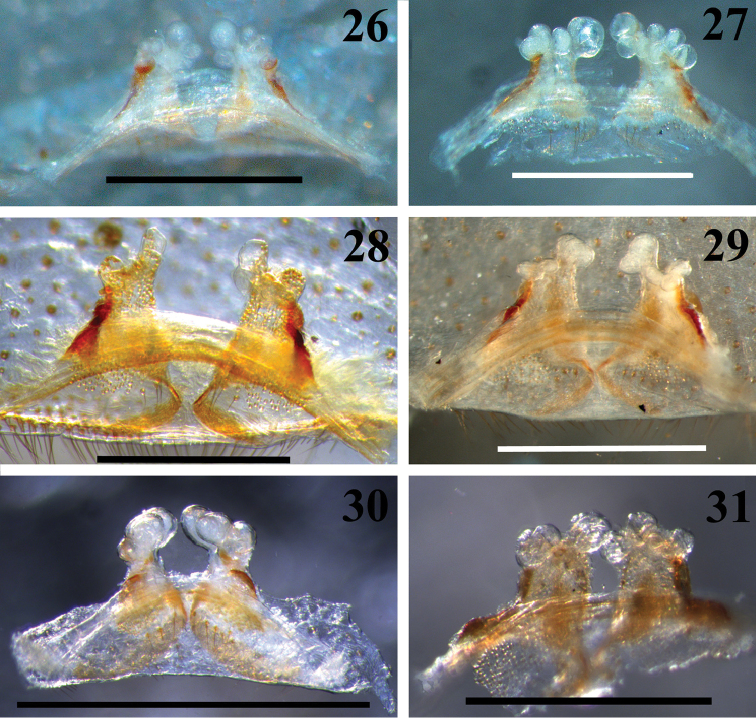
*Loxosceles amazonica*, seminal receptacles. **26** Martins, state of Rio Grande do Norte, Brazil (MNRJ 6942) **27** São Raimundo Nonato, state of Piauí, Brazil (MNRJ 6927, ref. GSB11A-17) **28** Serra Negra do Norte, ESEC Seridó, state of Rio Grande do Norte, Brazil (MNRJ 6928, ref. Ser 8) **29** Açu, FLONA de Açu, state of Rio Grande do Norte, Brazil (MNRJ 6931) **30** Santa Quitéria, state of Ceará, Brazil (MNRJ 6952) **31** Macaíba, state of Rio Grande do Norte, Brazil (MNRJ 6949). Scale bars: **27–29** 1 mm; **26, 30–31** 0.5 mm.

**Figures 32–35.**
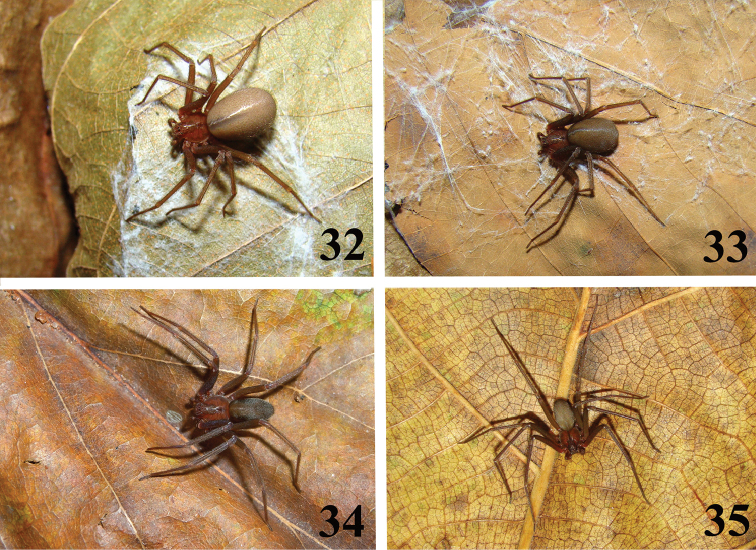
*Loxosceles amazonica*, habitus. **32–34** Female **32** Martins, state of Rio Grande do Norte, Brazil **33** Açu, FLONA de Açu, state of Rio Grande do Norte, Brazil **34** Macaíba, state of Rio Grande do Norte, Brazil **35** Male. Açu, FLONA de Açu, state of Rio Grande do Norte, Brazil (MNRJ 6936). Photos C. S. Fukushima.

**Figures 36–39.**
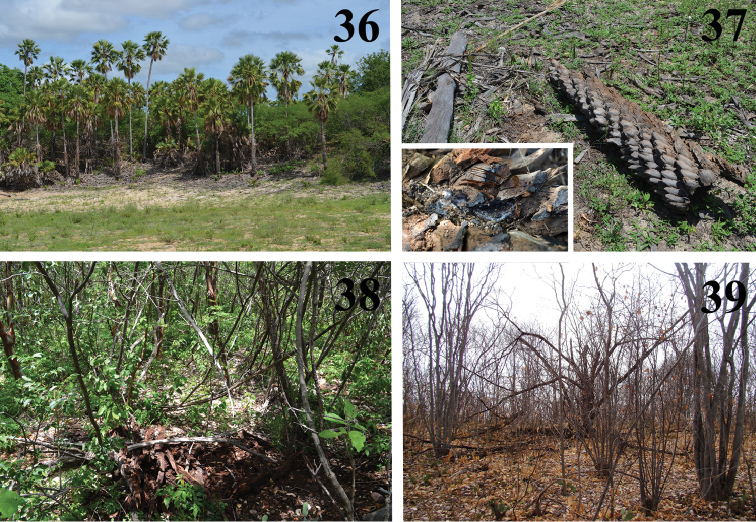
*Loxosceles amazonica* habitats in FLONA de Açu, Açu, state of Rio Grande do Norte, Brazil **36** Carnaúba trees **37** fallen Carnaúba tree, in detail web of *L. amazonica* **38** caatinga vegetation in rainy season **39** caatinga vegetation in dry season. Photos C. S. Fukushima.

**Figures 40–47.**
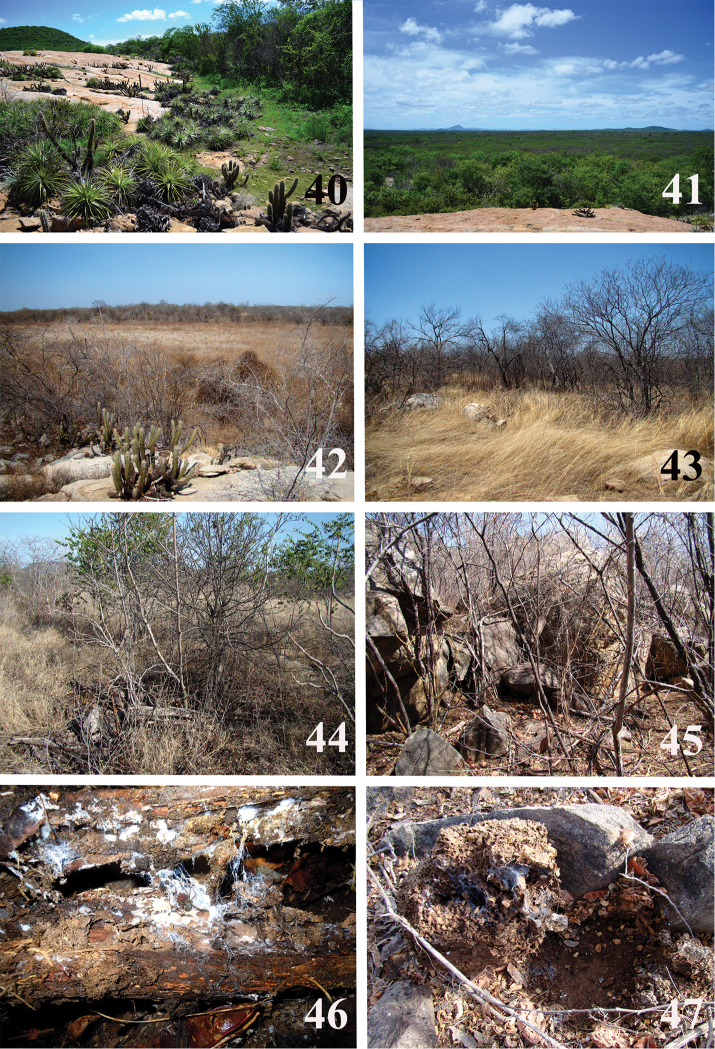
*Loxosceles amazonica* habitats in ESEC Seridó, Serra Negra do Norte, state of Rio Grande do Norte, Brazil. **40** large rocky outcrops **41** hyper-xerophilous, arboreal-shrubby caatinga in rainy season **42** dry temporary lagoon **43** grass areas over neosoil **44** fallen dead tree trunk in shaded area **45** small rocky outcrops **46** web of *L. amazonica* inside rotten tree trunk **47** web of *L. amazonica* inside termite nest. Photos C. S. Fukushima.

**Figures 48–51.**
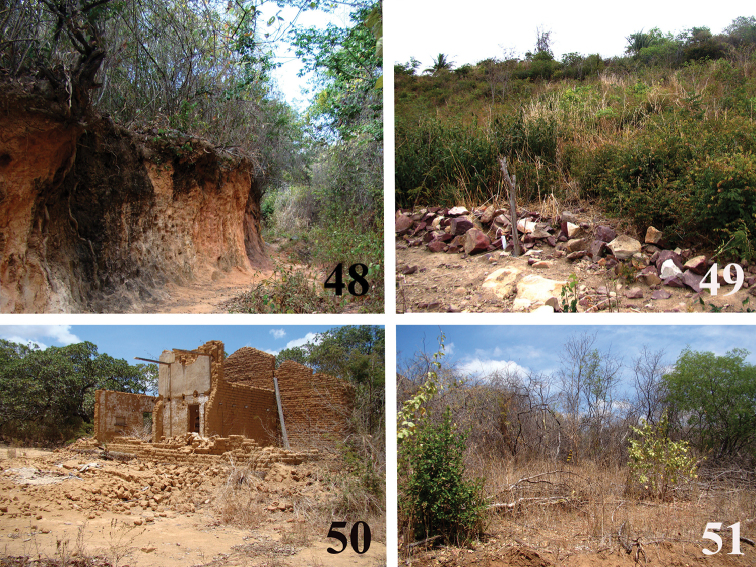
*Loxosceles amazonica* habitats in Martins, state of Rio Grande do Norte, Brazil **48** ravine in a humid area near town **49** under rocks at Mirante-Casa de Pedra cave trail **50** under debris of old house in rural area **51** in caatinga vegetation close to Casa de Pedra cave. Photos C. S. Fukushima.

### Loxosceles willianilsoni sp. n.

Taxon classificationAnimaliaAraneaeSicariidae

http://zoobank.org/DE5FF5FD-1637-461A-ACBD-93A670CC6E1F

Figs 52–55 , 56–57 , 58–61 , 62–65 , 66–69 , 78–79

#### Material examined

**(Table 3).** Male holotype (MNRJ 6953) and female paratype (MNRJ 6954), BRAZIL: *Rio Grande do Norte*, Martins, Casa de Pedra cave (06°05'S, 37°55'W), C. S. Fukushima col., 2014.

#### Other material examined

**(Table 3).** Casa de Pedra cave (06°05'S, 37°55'W), 319 m a.s.l., 1 female, A. P. L. Giupponi col., 2014, ref. A100 (MNRJ 6955); 1 female, N. M. Gonçalves col., 2014, ref. N60 (MNRJ 6956); 1 female, N. M. Gonçalves col., 2014, ref. N63 (MNRJ 6957); 1 female, C. S. Fukushima col., 2014, ref. C92 (MNRJ 6958); 1 female, C. S. Fukushima col., 2014, ref. C481 (MNRJ 6959); 1 male, N. M. Gonçalves col., 2014, ref. N59 (MNRJ 6960); 1 male, A. P. L Giupponi col., 2014, ref. A107 (MNRJ 6961); 1 male, C. S. Fukushima col., ref. C76 (MNRJ 6962); 1 male, K. C. T. Riciluca col., 2014, ref. K33 (MNRJ 6963); 1 male, A. P. L. Giupponi col., 2014, ref. A102 (MNRJ 6964); 1 male, C. S. Fukushima col., 2014, ref. C64 (MNRJ 6965); 1 male, C. S. Fukushima col., 2014, ref. C72 (MNRJ 6966), 1 female, C. S. Fukushima col, 2014, ref. C479 (MNRJ 6951).

#### Diagnosis.

**Figures 52–55.**
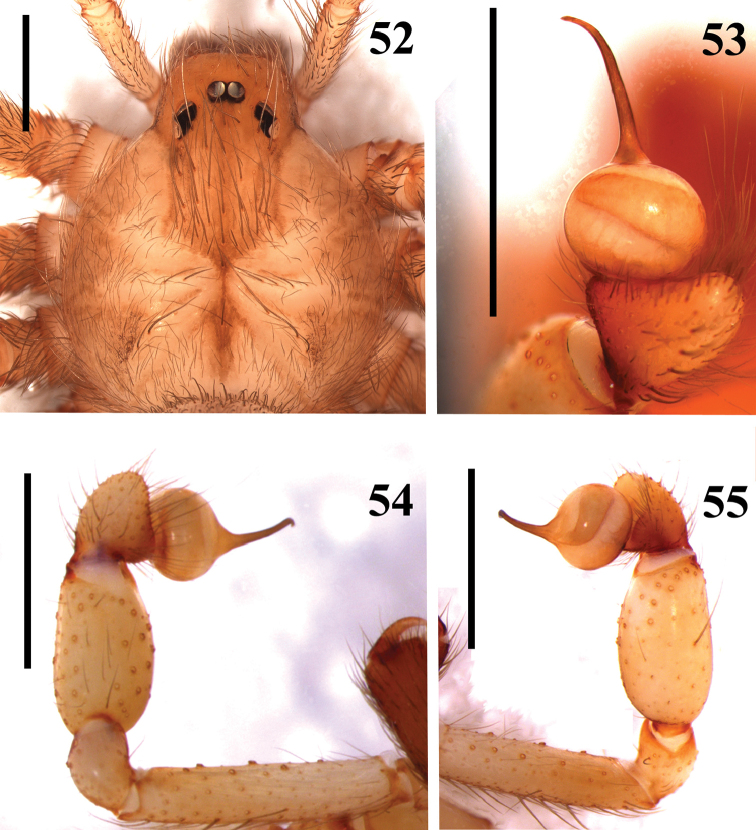
*Loxosceles willianilsoni* sp. n., male holotype (MNRJ 6953). **52** carapace **53–55** left palpal bulb **53** dorsal **54** prolateral **55** retrolateral. Scale bar 1mm.

Males of *Loxosceles willianilsoni* sp. n. resemble those of *L. amazonica, L. rufescens*, *L. bentejui*, *L. foutadjalloni*, *L. guayota*, *L. hupalupa*, *L. lacta*, *L. mahan*, *L. tazarte*, *L. tibicena*, and *L. muriciensis* sp. n. by incrassated palpal tibia, longer than cymbium (Fig. 54). They differ from those of *L. hupalupa*, *L. mahan* and *L. tazarte* by having shorter embolus (Fig. 54), and entire pars cephalica as well as carapace border dark brown (Fig. 52), best seen in live specimens. From those of *L. amazonica, L. rufescens*, *L. bentejui*, *L. foutadjalloni*, *L. guayota*, *L. lacta*, *L. tibicena*, and *L. muriciensis* sp. n. they can be distinguished by having straight embolus with a strong curvature on its apex (Fig. 53). Additionally, males of *L. willianilsoni* sp. n. differ from those of all these species except *L. foutadjalloni*, *L. guayota*, and *L. muriciensis* sp. n. by having leg I at least eight times as long as carapace (Table 1). Females of *L. willianilsoni* sp. n. resemble females of *L. amazonica, L. rufescens*, *L. bentejui*, *L. foutadjalloni*, *L. hupalupa*, *L. lacta*, *L. mahan*, *L. tazarte*, *L. tibicena*, and *L. muriciensis* sp. n. by having spermathecae with large seminal receptacles and dark sclerotized lateral bands (Fig. 57). Females of *L. willianilsoni* sp. n. can be distinguished from all these species by the combination of the following characters: spermathecae with dark sclerotized lateral bands almost reaching their apex, which has no lobes and no constriction forming a neck (Figs 57–61), leg I at least 6.5 times as long as carapace (Table 2). Additionally, *L. willianilsoni* sp. n. males and females can be distinguished from *L. mahan*, *L. tazarte*, *L. bentejui*, *L. guayota*, *L. tibicena* and *L. hupalupa* by lacking a conspicuous dark V-mark posteriorly on pars cephalica.

#### Description.

*Male* holotype (MNRJ 6953). Total length 7.39. Carapace 3.16 long, 2.74 wide. Eye sizes and interdistances: ALE 0.15, PME 0.21, PLE 0.18, PME-PLE 0.02, PME-ALE 0.15; clypeus 0.26. Leg formula II, I, IV, III. Legs length: leg I: femur 7.47, patella 0.98, tibia 8.37, metatarsus 8.85, tarsus 1.77, total 27.44; II: 8.29, 1.11, 9.88, 10.95, 1.85, 32.08; III: 6.40, 1.09, 6.23, 7.64, 1.30, 22.66; IV: 7.12, 1.05, 7.08, 8.38, 1.52, 26.15. Palp: femur 1.46 long, 0.31 wide; patella 0.49 long, 0.33 wide; tibia 0.88 long, 0.48 wide; cymbium 0.43 long, 0.42 wide. Labium 0.71 long, 0.38 wide. Sternum 1.78 long, 1.50 wide. Femur I 2.4 times as long, tibia I 2.7 times as long and leg I 8.7 as long as carapace. Palpal femur four times longer than wide, tibia 1.8 times longer than wide, cymbium oval (Fig. 54). Bulb suboval and approximately same size as cymbium. Embolus straight, with a strong curvature on apex, approximately 1.3 times longer than bulb length in retrolateral view, without carina (Fig. 53). Cephalic region of carapace covered by many long setae (Fig. 52). Entire pars cephalica as well as carapace border dark brown (Fig. 52). Legs and palps light brown, covered by short greyish setae on the femora and patellae (Fig. 64). Endites, coxae and sternum light brown. Labium dark brown.

*Female paratype* (MNRJ 6954): As in male, except: Total length 8.72. Carapace 2.99 long, 2.39 wide. Eye sizes and interdistances: ALE 0.14, PME 0.17, PLE 0.16, PME-PLE 0.02, PME-ALE 0.19; clypeus 0.35. Leg formula II, I, IV, III. Legs length: leg I: femur 5.25, patella 1.17, tibia 5.93, metatarsus 5.88, tarsus 1.24, total 19.47; II: 5.96, 1.14, 6.40, 6.32, 1.50, 21.32; III: 4.76, 1.00, 4.22, 4.80, 1.19, 15.97; IV: 5.32, 1.15, 4.89, 5.96, 1.40, 18.72. Palp: femur 0.98 long, 0.21 wide; patella 0.28 long, 0.25 wide; tibia 0.70 long, 0.20 wide; tarsus 1.06 long, 0.16 wide. Labium 0.53 long, 0.44 wide. Sternum 1.63 long, 1.38 wide. Femur I 1.8 times as long, tibia I 2.0 times as long and leg I 6.5 as long as carapace. Palpal femur 4.7 times longer than wide, tibia 3.5 longer than wide, tarsus not incrassate. Spermathecae with enlarged seminal receptacles; without transversal plate; and presence of dark sclerotized lateral bands almost reaching the apex (Fig. 57). Palps pale brown, except by darker tibiae and metatarsi. Endites pale brown.

#### Etymology.

This species is named after the biology student Willianilson Pessoa, in honor of his friendship and support during expeditions in Rio Grande do Norte. This name is masculine in gender.

#### Natural history.

Specimens were found inside Casa de Pedra cave walking on walls, in webs inside wall cracks or under loose stones on the cave ground. This calcarian cave is very large regarding regional patterns and has turistic use (Ferreira et al. 2010). Apparently, specimens of *L. willianilsoni* sp. n. are found only inside the cave.

**Figures 56–57.**
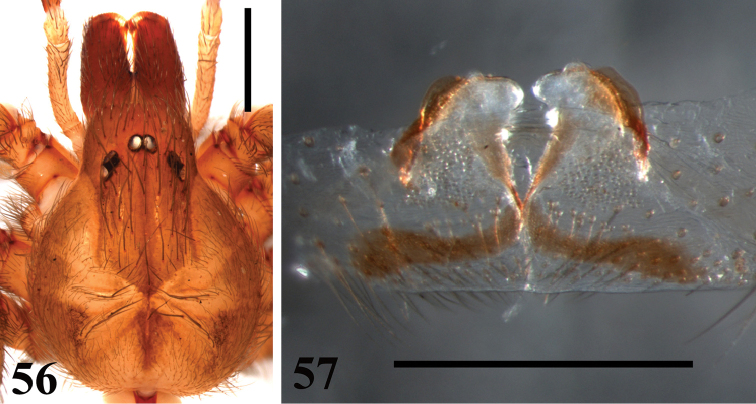
*Loxosceles willianilsoni* sp. n., female paratype (MNRJ 6954). **56** carapace **57** seminal receptacles. Scale bar: 1 mm.

**Figures 58–61.**
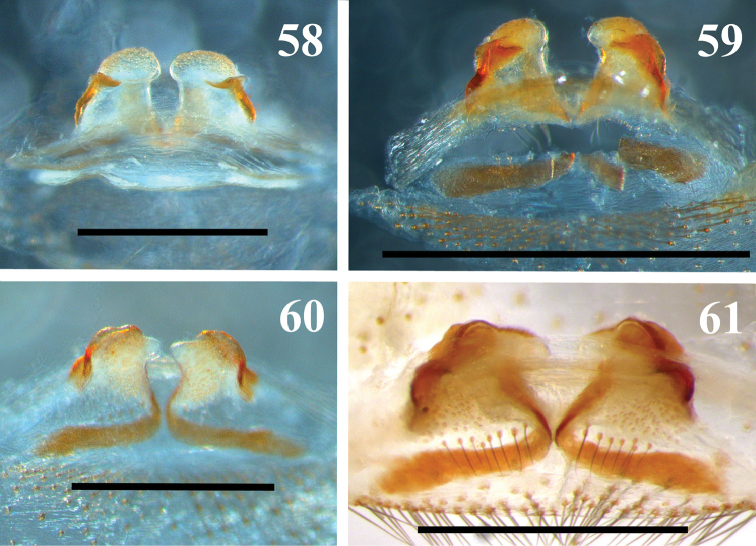
*Loxosceles willianilsoni* sp. n., seminal receptacles variation. **58** MNRJ 6957 **59** MNRJ 6956 **60** MNRJ 6959 **61** MNRJ 6951. Scale bars: **58–60** 1 mm; **61** 0.5 mm.

**Figures 62–65.**
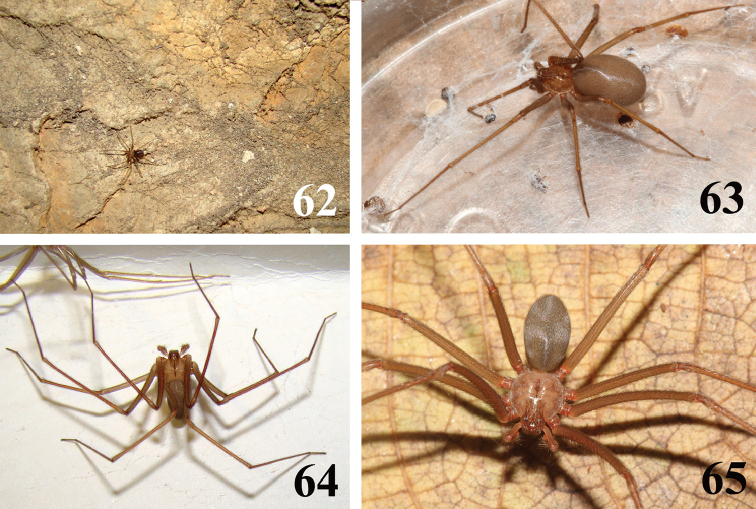
*Loxosceles willianilsoni* sp. n., habitus. **62** specimen walking inside Casa de Pedra cave **63** female **64** male **65** carapace pattern, male. Photos **62, 64** C. S. Fukushima; **63, 65** R. Bertani.

**Figures 66–69.**
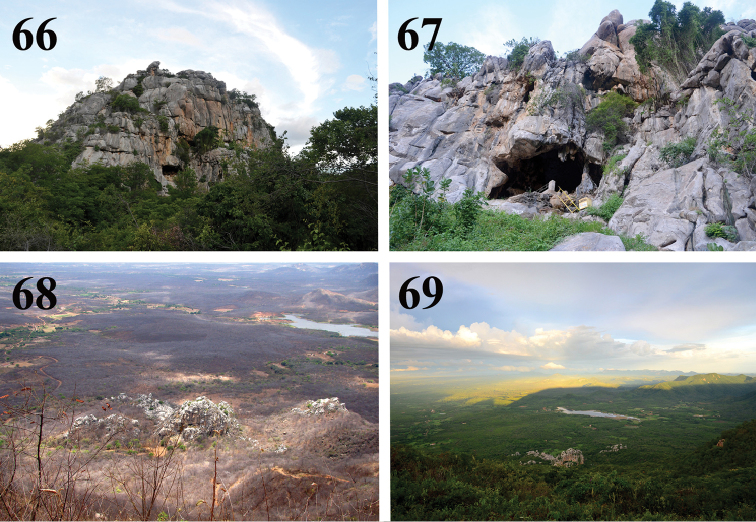
*Loxosceles willianilsoni* sp. n. habitat in Martins, state of Rio Grande do Norte, Brazil **66** Casa de Pedra cave **67** entrance of the cave **68–69** caatinga vegetation surrounding the cave **68** dry season **69** rainy season. Photos C. S. Fukushima.

### Loxosceles muriciensis sp. n.

Taxon classificationAnimaliaAraneaeSicariidae

http://zoobank.org/CC85E3A6-44F7-4C7C-BCBD-EA9002A7309F

Figs 70–73 , 74–75 , 76–77 , 78–79

#### Material examined

**(Table 3).** Male holotype (MNRJ 6967) and female and male paratypes (MNRJ 6968), BRAZIL: *Alagoas*, Murici, Estação Ecológica de Murici (9°15'S, 35°48'W), 23.1°C, 84% URA, under the bark of a large burnt tree, R. Bertani, D. R. M. Ortega and R. H. Nagahama col., 13 August 2006.

#### Diagnosis.

**Figures 70–73.**
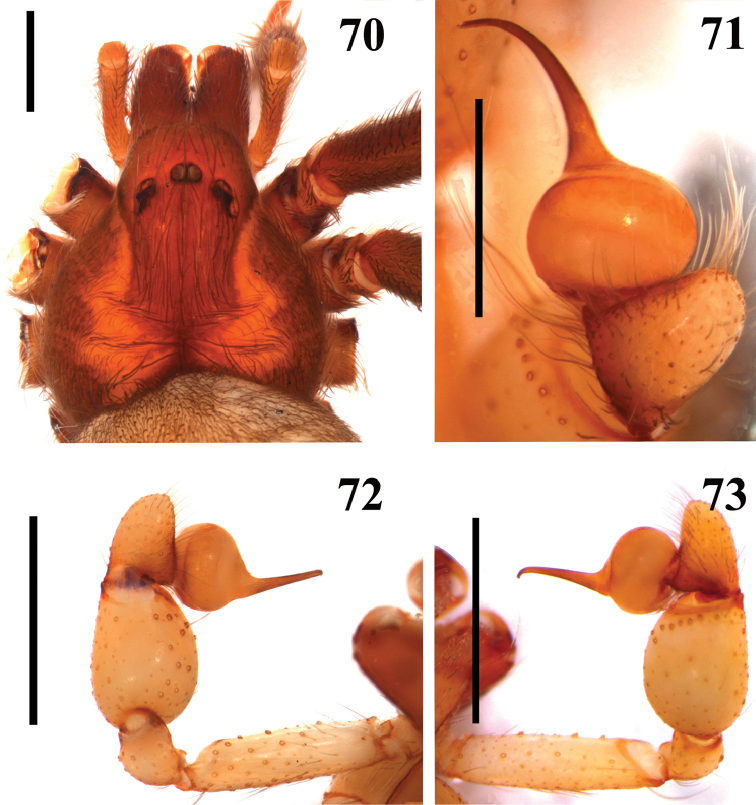
*Loxosceles muriciensis* sp. n., male holotype. **70** carapace **71–73** right palpal bulb **71** dorsal (mirrored) **72** prolateral (mirrored) **73** retrolateral (mirrored). Scale bars: **70, 72–73** 1mm;**71** 0.5mm.

Males of *L. muriciensis* sp. n. resemble those of *L. amazonica, L. rufescens*, *L. bentejui*, *L. foutadjalloni*, *L. guayota*, *L. hupalupa*, *L. lacta*, *L. mahan*, *L. tazarte*, *L. tibicena* and *L. willianilsoni* sp. n. by incrassated palpal tibia, longer than cymbium (Fig. 72). Males differ from those of *L. hupalupa*, *L. mahan* and *L. tazarte* by having shorter embolus (Fig. 72), and entire pars cephalica as well as carapace border dark brown (Fig. 70), best seen in live specimens. Males of *L. muriciensis* sp. n. differ from those of *L. amazonica, L. rufescens*, *L. bentejui*, *L. foutadjalloni*, *L. guayota*, *L. lacta*, *L. tibicena*, and *L. willianilsoni* sp. n. by having straight embolus with a mild curvature on apex, forming a hook (Fig. 71). Additionally, males of *Loxosceles muriciensis* sp. n. differ from males of all these species except *L. foutadjalloni*, *L. guayota* and *L. willianilsoni* sp. n. by having leg I at least eight times as long as carapace (Table 1). Females of *L. muriciensis* sp. n. resemble those of *L. amazonica, L. rufescens*, *L. bentejui*, *L. foutadjalloni*, *L. hupalupa*, *L. lacta*, *L. mahan*, *L. tazarte*, *L. tibicena*, and *L. willianilsoni* sp. n. by having spermathecae with large seminal receptacles and dark sclerotized lateral bands (Fig. 75). Females of *L. muriciensis* sp. n. can be distinguished from all these species by the following combination of characters: spermathecae with dark sclerotized lateral bands almost reaching their apex, which has two well-developed lobes, and no constriction forming a neck (Fig. 75); leg I more than five times as long as carapace (Table 2). Additionally, *L. muriciensis* sp. n. males and females can be distinguished from *L. mahan*, *L. tazarte*, *L. bentejui*, *L. guayota*, *L. tibicena* and *L. hupalupa* by lacking a conspicuous dark V-mark posteriorly on pars cephalica.

#### Description.

*Male* holotype (MNRJ 6967). Total length 5.46. Carapace 2.21 long, 2.10 wide. Eye sizes and interdistances: ALE 0.12, PME 0.16, PLE 0.16, PME-PLE 0.02, PME-ALE 0.12; clypeus 0.30. Leg formula II, I, IV, III. Legs length: leg I: femur 4.73, patella 0.90, tibia 5.20, metatarsus 5.65, tarsus 1.42, total 17.9; II: 5.15, 0.95, 5.13, 6.39, 1.45, 19.07; III: 4.21. 0.70. 3.73. 4.37. 0.93. 13.94; IV: 4.77. 0.69. 4.55. 5.55. 1.15. 16.71. Palp: femur 1.12 long, 0.30 wide; patella 0.46 long, 0.35 wide; tibia 0.70 long, 0.55 wide; cymbium 0.50 long, 0.35 wide. Labium 0.49 long, 0.33 wide. Sternum 1.23 long, 1.16 wide. Femur I 2.2 times as long, tibia I 2.4 times as long and leg I 8.1 as long as carapace. Palpal femur 3.7 times longer than wide, tibia 1.3 times longer than wide, cymbium oval (Fig. 72). Bulb suboval and larger than cymbium. Embolus straight, with a mild curvature on apex, approximately 1.6 times longer than bulb length in retrolateral view, without carina (Fig. 71). Cephalic region of carapace covered by many long setae (Fig. 70). Entire pars cephalica as well as carapace border dark brown (Fig. 70). Legs and palps light brown, covered by short greyish setae on the femora and patellae. Endites, coxae and sternum light brown. Labium dark brown.

*Female paratype* (MNRJ 6968): As in male, except: Total length 8.65. Carapace 2.98 long, 2.80 wide. Eye sizes and interdistances: ALE 0.15, PME 0.21, PLE 0.20, PME-PLE 0.05, PME-ALE 0.17; clypeus 0.40. Leg formula II, I, IV, III. Legs length: leg I: femur 4.51, patella 1.13, tibia 4.50, metatarsus 4.35, tarsus 1.45, total 15.94; II: 5.05, 1.06, 5.33, 3.41, 1.30, 16.15; III: 4.25, 1.05, 3.55, 4.30, 1.02, 14.17; IV: 4.55, 0.62, 4.50, 3.45, 1.22, 14.34. Palp: femur 1.20 long, 0.25 wide; patella 0.37 long, 0.31 wide; tibia 0.71 long, 0.25 wide; tarsus 1.07 long, 0.17 wide. Labium 0.58 long, 0.50 wide. Sternum 1.84 long, 1.40 wide. Femur I 1.5 times as long, tibia I 1.5 times as long and leg I 5.3 as long as carapace. Palpal femur 4.8 times longer than wide, tibia 2.8 longer than wide, tarsus not incrassate. Spermathecae with enlarged seminal receptacles; without transversal plate, lacking a constriction near apex forming a neck; presence of two well-developed lobes on apex and dark sclerotized lateral bands almost reaching apex (Fig. 75). Palps brown, except by pale patellae and femora. Endites pale brown.

#### Etymology.

The specific name refers to the type locality, Estação Ecológica de Murici, state of Alagoas, Brazil and is neutral in gender.

#### Natural history.

The few specimens of *L muriciensis* sp. n. were found inside a burnt tree in an Atlantic rainforest conservation unit in the state of Alagoas. The ESEC Murici is one of the last and largest remnants of the northeastern Atlantic rainforest and it is inserted in a biodiversity hotspot known as the “Pernambuco Endemism Center” (Nemésio and Santos Junior 2014).

**Figures 74–75.**
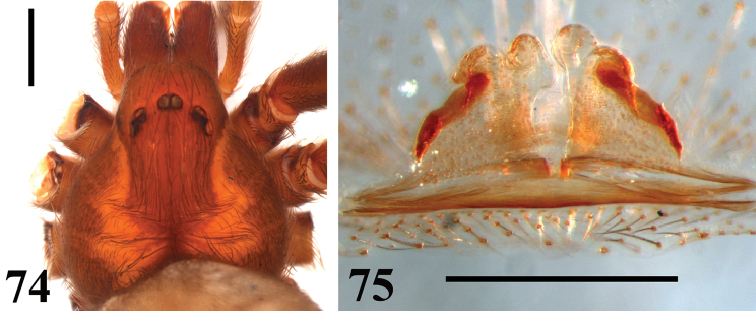
*Loxosceles muriciensis* sp. n., female paratype. **74** carapace **75** seminal receptacles. Scale bars: 1 mm.

**Figures 76–77.**
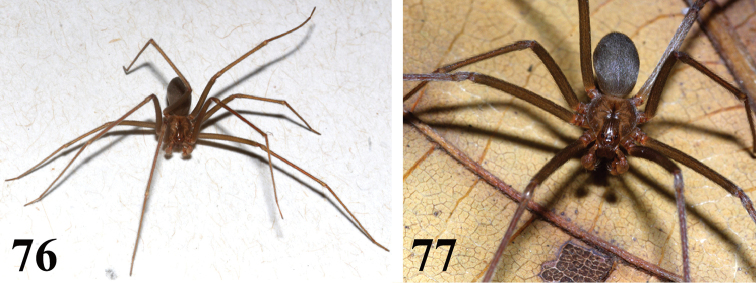
*Loxosceles muriciensis* sp. n. male holotype, habitus. **76** overall aspect **77** carapace pattern. Photos R. Bertani.

**Figures 78–79.**
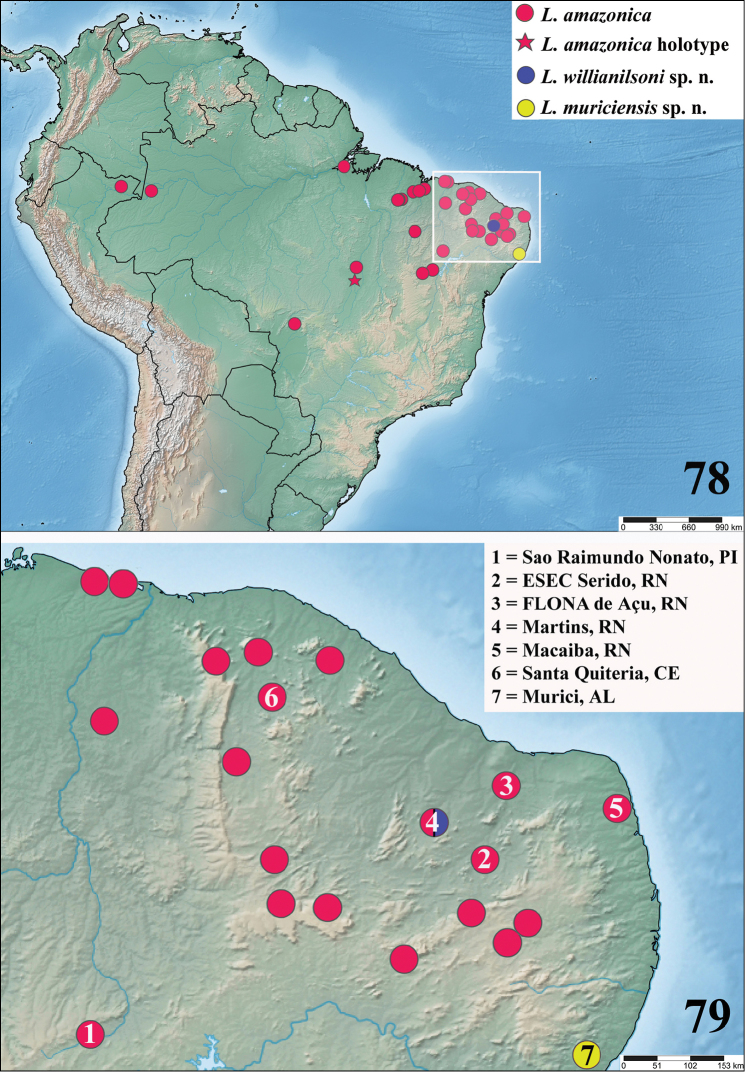
**78** Map showing records of *L. amazonica*, *L. willianilsoni* sp. n. and *L. muriciensis* sp. n. Area inside rectangle represented on Figure 79. Records of *L. amazonica* include also those from Azevedo et al. (2014), Gertsch (1967) and Silveira (2015). **79** Expanded map showing the records of the illustrated specimens of *L. amazonica*, *L. willianilsoni* sp. n. and *L. muriciensis* sp. n.

**Table 1.** *Loxosceles* spp. of *rufescens* group, males. Carapace and leg I measurements. Data from (1) Gertsch (1967), (2) Lotz (2012), (3) Planas and Ribera (2015). Legs differentiated by less than 0.5 mm are in bold. AL = state of Alagoas, AM = state of Amazonas, CE = state of Ceará, PI = state of Piauí, RN = state of Rio Grande do Norte.

Taxon	Locality	Specimen	Carapace	Leg I	Leg I / Carapace	Leg Formula
*L. amazonica* ^1^	Gurupá (AM), Brazil	paratype	4	19.5	4.88	2, **4**, **1**, 3
*L. amazonica*	FLONA Açu (RN), Brazil	MNRJ 6936	3.87	22.11	5.72	2, **4**, **1**, 3
*L. amazonica*	ESEC Seridó (RN), Brazil	MNRJ 7303	3.26	17.57	5.39	2, 4, 1, 3
*L. amazonica*	Martins (RN), Brazil	MNRJ 6947	3.12	16.94	5.43	2, 4, 1, 3
*L. amazonica*	Macaíba (RN), Brazil	MNRJ 6948	2.86	15.95	5.58	**2**, **4**, 1, 3
*L. amazonica*	São Raimundo Nonato (PI), Brazil	MNRJ 6927	2.62	16.39	6.25	2, **4**, **1**, 3
*L. amazonica*	Santa Quitéria (CE), Brazil	MNRJ 6950	2.76	20.81	7.54	2, **4**, **1**, 3
*L. willianilsoni* sp. n.	Martins (RN), Brazil	holotype	3.16	27.44	8.69	2, 1, 4, 3
*L. muriciensis* sp. n.	Murici (AL), Brazil	holotype	2.21	17.9	8.12	2, 1, 4, 3
*L. rufescens* ^1^	Rome, Italy	AMNH	3	20.1	6.70	2, **4**, **1**, 3
*L. foutadjalloni* ^2^	Guinea	lectotype	4	45.9	11.48	2, 1, 4, 3
*L. mahan* ^3^	Canary Islands	holotype	2.89	17.37	6.01	2, **4**, **1**, 3
*L. tazarte* ^3^	Canary Islands	holotype	2.34	15.42	6.59	2, **1**, **4**, 3
*L. bentejui* ^3^	Canary Islands	holotype	2.91	20.63	7.09	2, 1, 4, 3
*L. tibicena* ^3^	Canary Islands	holotype	2.63	20.19	7.68	2, 4, 1, 3
*L. guayota* ^3^	Canary Islands	holotype	3.62	34.78	9.61	2, 1, 4, 3
*L. hupalupa* ^3^	Canary Islands	holotype	2.51	19.51	7.77	2, 4, 1, 3

**Table 2.** *Loxosceles* spp. of *rufescens* group, females. Carapace and leg I measurements. Data from (1) Gertsch (1967), (2) Lotz (2012), (3) Planas and Ribera (2015). Legs differentiated by less than 0.5 mm are in bold. * = Legs 2 and 4, and legs 4 and 1 have difference in length less than 0.5 mm. AL = state of Alagoas, CE = state of Ceará, MT = state of Mato Grosso, PI = state of Piauí, RN = state of Rio Grande do Norte.

Taxon	Locality	Specimen	Carapace	Leg I	Leg I / Carapace	Leg Formula
*L. amazonica*	Santa Isabel (MT), Brazil	holotype	4.17	19.04	4.57	2, **4**, **1**, 3
*L. amazonica*	Açu (RN), Brazil	MNRJ 6933	3.82	17.32	4.53	2, **4**, **1**, 3
*L. amazonica*	Serra Negra do Norte (RN), Brazil	MNRJ 6928	3.89	17.60	4.52	**2**, **4**, **1**, 3*
*L. amazonica*	Martins (RN), Brazil	MNRJ 6940	3.83	17.63	4.60	2, **4**, **1**, 3
*L. amazonica*	Macaíba (RN), Brazil	MNRJ 6949	3.45	14.06	4.08	Missing legs 3 and 4
*L. amazonica*	São Raimundo Nonato (PI), Brazil	MNRJ 6927	3.08	12.48	4.06	**2**, **4**, **1**, 3*
*L. amazonica*	Santa Quitéria (CE), Brazil	MNRJ 6952	2.86	16.56	5.79	2, 4, 1, 3
*L. willianilsoni* sp. n.	Martins(RN), Brazil	paratype	2.99	19.47	6.52	2, 1, 4, 3
*L. muriciensis* sp. n.	Murici (AL), Brazil	paratype	2.98	15.94	5.34	2, 1, 4, 3
*L. rufescens* ^1^	Alto Douro, Portugal	AMNH	3.2	15.4	4.81	2, 4, 1, 3
*L. foutadjalloni* ^2^	Guinea	paralectotype	3.9	26.8	6.87	2, 1, 4, 3
*L. mahan* ^3^	Canary Islands	paratype	2.97	12.97	4.37	**2**, **4**, 1, 3
*L. tazarte* ^3^	Canary Islands	paratype	2.88	14.65	5.09	2, **4**, **1**, 3
*L. bentejui* ^3^	Canary Islands	paratype	3.35	16.78	5.01	2, **4**, **1**, 3
*L. tibicena* ^3^	Canary Islands	paratype	3.35	18.43	5.50	2, **4**, **1**, 3
*L. hupalupa* ^3^	Canary Islands	paratype	3.71	23.09	6.22	Missing leg 4

**Table 3.** Localities of all the material studied. F = female, J = juvenile, M= male, MJ= immature male.

Species	Quantity	Number	Locality	Coordinates
*L. amazonica*	1M, 1F, 11J	MNRJ 6927	PARNA Serra da Capivara, São Raimundo Nonato, Piauí, Brazil	[9°00'S, 42°41'W]
	5M, 2F	MNRJ 6928	ESEC Seridó, Serra Negra do Norte, Rio Grande do Norte, Brazil	(6°34'S, 37°15'W)
	1F	MNRJ 6929		
	1F	MNRJ 6930		
	1M	MNRJ 7303		
	1F	MNRJ 6931	FLONA de Açu, Açu, Rio Grande do Norte, Brazil	(5°34'S, 36°56'W)
	1F	MNRJ 6932		
	1F	MNRJ 6933		
	1F	MNRJ 6934		
	1M	MNRJ 6935		
	1M	MNRJ 6936		
	1M	MNRJ 6937		
	1M	MNRJ 6938		
	1M	MNRJ 6939		
	1F	MNRJ 7305		
	1F	MNRJ 6940	Martins, Rio Grande do Norte, Brazil	(6°04'S, 37°54'W)
	1F	MNRJ 6941		
	1F	MNRJ 6942		
	1F	MNRJ 6943		
	1F	MNRJ 6944		
	1M	MNRJ 6945		
	1M	MNRJ 6946		
	1M	MNRJ 6947		
	1M	MNRJ 7306		
	1F	MNRJ 7304		
	1M	MNRJ 6948	Macaíba, Rio Grande do Norte, Brazil	(5°53'S, 35°21'W)
	1F	MNRJ 6949		
	1M, 1MJ	MNRJ 6950	Santa Quitéria, Ceará, Brazil	(4°19'S, 40°09'W)
	1M, 1F, 9J	MNRJ 6952		
*L. willianilsoni* sp. n.	1M	MNRJ 6953	Casa de Pedra cave, Martins, Rio Grande do Norte, Brazil	(06°05'S, 37°55'W)
	1F	MNRJ 6954		
	1F	MNRJ 6955		
	1F	MNRJ 6956		
	1F	MNRJ 6957		
	1F	MNRJ 6958		
	1F	MNRJ 6959		
	1M	MNRJ 6960		
	1M	MNRJ 6961		
	1M	MNRJ 6962		
	1M	MNRJ 6963		
	1M	MNRJ 6964		
	1M	MNRJ 6965		
	1M	MNRJ 6966		
	1F	MNRJ 6951		
*L. muriciensis* sp. n.	1M	MNRJ 6967	Murici, Alagoas, Brazil	(9°15'S, 35°48'W)
	1F, 1M	MNRJ 6968		

## Discussion

In his revision of the South American *Loxosceles* species, Gertsch (1967) proposed four species groups for the thirty species he recognized. The only group with a single species is *amazonica* with the species *L. amazonica* described in the same paper (Gertsch 1967). This author approximated *L. amazonica* to the *gaucho* group due to the carapace marked with dark lateral bands and some incrassated segments of male palps. On the other hand, the presence of spermathecae with free receptacles with rounded lobes, not closely tied by a transverse band, resembles *laeta* species (Gertsch 1967). Despite *L. amazonica* having characteristics of both South American groups *gaucho* and *laeta*, in some genitalic features it closely resembles species of the *rufescens* group from the Paleartic fauna (Gertsch 1967). Due to these special characteristics, *L. amazonica* was considered to have group status by Gertsch (1967).

After Gertsch’s revision (1967), only scattered descriptions of new species of *Loxosceles* were published. A more embracing work was done by Binford et al. (2008), which proposed the first phylogenetic relationship hypothesis concerning representative *Loxosceles* species. In that work, besides morphological similarity, a molecular proximity was detected between *L. amazonica* and *L. rufescens* (Binford et al. 2008). The ubiquitous species *L. rufescens*, associated or not with the Chinese species *L. lacta*, was presented as the sister-group of *L. amazonica* in analyses with different types and combinations of datasets (Binford et al. 2008). The authors considered two possible explanations for the strong evidence of a close relationship between these species. In one explanation, the *rufescens* lineage would be old, with the ancestors of both species pre-dating the split of the continents; in the other, the lineage would be younger and it was suggested to be a natural dispersion from South America to Africa after the continent split occurred. According to the authors, the great genetic divergence found between *L. amazonica* and *L. rufescens* and the species diversity of the *rufescens* group in the Old World makes the human-mediated transportation explanation unlikely (Binford et al. 2008). However, the divergence date between *L. amazonica* and *L. rufescens* estimated by Binford et al. (2008) is too young for the presence of the most recent ancestor on Gondwana. Binford et al. (2008) also stated that the current range of *L. amazonica* and *L. rufescens*, northeastern South America and North Africa respectively, is compatible either with the Gondwana ancestor explanation or with dispersal through temporary land corridors after continental split. Thus, the distinction between ancient vicariance and more recent dispersion to explain the relationship of both species would require the inclusion of more species of these related areas in a more extensive analysis (Binford et al. 2008).

A more detailed study of the diversity of the northwestern African *Loxosceles* species and new molecular phylogenetic analyses including *L. rufescens* and *L. amazonica* was done by Duncan et al. (2010). Once again, *L. amazonica* was recovered in the clade including the northwestern African *Loxosceles* species. However, there was no agreement that *L. amazonica* was the sister-group of the monophyletic *L. rufescens* lineage nor the basal taxa of the northwestern African clade. The lack of resolution inside the northwestern African clade, the existence of African male specimens very similar morphologically to *L. amazonica* and the fact that the most recent common ancestor of *L. amazonica* and *L. rufescens* was found by Binford et al. (2008) to be too young to be explained by Gondwanan vicariance were considered by Duncan et al. (2010) to indicate that *L. amazonica* is derived from within northwest Africa *Loxosceles* and dispersed recently from one continent to other. They proposed that the split of the continents did not influence the distribution of the common ancestor *L. amazonica* and *L. rufescens* (Duncan et al. 2010). They considered *L. amazonica* as a species that can be easily introduced by human transport and suggested the trade between Brazil and Africa in 16^th^ century could explain the dispersal of *L. amazonica* from Africa to South America (Duncan et al. 2010). They also considered the absence of other species related to *L. amazonica* in South America as further evidence supporting an African origin of this species.

The discovery of two new species, herein described, closely related to *L. amazonica* in northwestern Brazil, throw a new light on this discussion. It is very unlikely that *L. amazonica* came from Africa about 500 years ago and in so little time speciated into two more different species. Another point that contradicts the argument that *L. amazonica* was introduced in South America is the large distribution of the species (Fig. 78). It is very improbable that such a reclusive spider would disperse to many natural localities throughout northwestern Brazil in such a short period of time, reaching remote localities in central western Brazil such as the type locality, an indigenous village difficult to access even nowadays. Furthermore, specimens of *L. amazonica* as well specimens of *L. willianilsoni* sp. n. and *L. muriciensis* sp. n. were found in natural environments (Figs 39–47, 66) inside and outside four Conservation Units in three Brazilian states. Moreover, if *L. amazonica* is an invasive species as proposed by Duncan et al. (2010), their presence in larger cities in southeastern and southern Brazil would also be expected, as invasive species are normally introduced by means of human activities and benefited by urban environments, normally forming large populations. Even though they can be found in disturbed environments in northwestern Brazil, they are found in natural conditions and are not found in urban areas in localities more to the South.

The question on the origin of *L. amazonica* and *L. rufescens* lineages is, therefore, open to discussion. A way to test the origin and evolution of *L. amazonica* lineage would be to collect *L. amazonica* specimens from different parts of northern, northwestern and central western Brazil as well as other South American countries, and determine the genetic divergence among the different populations.

As demonstrated by Duncan et al. (2010), the *amazonica* group is recovered in the middle of *rufescens* lineage. Therefore, it makes no sense to use the group name *amazonica*, and *L. amazonica*, *L. willianilsoni* sp. n. and *L. muriciensis* sp. n. should be referred as belonging to *rufescens* group.

## Supplementary Material

XML Treatment for Loxosceles amazonica

XML Treatment for Loxosceles willianilsoni

XML Treatment for Loxosceles muriciensis
